# The Attitude of Great Writers Towards Disease

**Published:** 1892-10-29

**Authors:** 


### The Attitude of Great Writers Towards Disease.

### XIV.—SIR THOMAS BROWNE.

Sir Thomas Browne holds an important place in the 17th century at the parting of two roads in the history of English medical science. Medicine owes him, it is true, no great discoveries. The learned physician, after a course of study embracing all that was to be learnt on the subject of his profession at home and abroad, from books and observation, was content to spend his days in the comparative obscurity of a country practice, and consecrate the time which could be spared from ministrations on the burgesses and poor of Norwich to the pursuit of letters. The value of Sir Thomas Browne's writings from a medical point of view lies in the circumstance that in him was combined the dying spirit of the ages of irresponsible guesswork and reverence for antiquity, with the new experimental methods inaugurated by Bacon, and crowned with such brilliant success in the discoveries of William Harvey. He is in touch with both old and new, and his writings reflect alternately the scholar's regard for the venerable dicta of classic authors and the shrewdness of the keen observer, vowed to accept nothing admitting of proof without personal investigation. That he should find it necessary to refute so many transparent absurdities is no less amazing than his tenacious hold on some of the fallacious opinions of his age, and gives a curious impression of the haziness of men's views about the simple facts "now taught by village dames."

It is strange, for instance, to hear this cautious and sceptical physician asserting "for my part I have ever believed, and do now know, that there are witches," and again "that phantasms appear often and do frequent cemeteries, charnel houses, and churches."

Yet scepticism prevailed in the long run over credulity, and his exact inquiry into the easy beliefs of the day went near to procure him among his contemporaries (most unjustly, as we shall see) the title of Atheist.

In his "Vulgar Errors" he dealt with a vast collection of theories, recipes, and traditions of all ages and nations. Such as admitted of experimental refutation were submitted to the test, and others beyond the reach of this satisfaction were even more convincingly demolished by argument. The properties of the loadstone, at that time credulously relied on for the most varied purposes furnish examples of both methods. He was much interested in the "method of curation lately delivered by Daniel Becherus of a young man of Spruceland that casually swallowed a knife about 10 inches long, which was cut out of his stomach, and the wound healed up; in which cure, to attract the knife to a convenient situation, there was applied a plaster made up with the powder of loadstone." Patients who casually swallow knives 10 inches long are not plentiful, but the doctor succeeds, without eye witness, in making even the unlearned doubtful of the efficacy of such a poultice. Again, he combats the received opinion that if two needles be touched with the same loadstone, and set in circles inscribed with the letters of the alphabet, the one needle will move in sympathy with the other, however far they may be separated. This time he can assure his readers that he has set up the whole apparatus in adjoining rooms, and proved its falsity, something, it may be guessed, to his own disappointment, and this makes him doubtful about a somewhat similar method of communicating with absent friends, viz., whether "if a permutation of flesh be made from one man's body to another, and about them both an alphabet be circumscribed, upon a time appointed, they may communicate at how great a distance soever" by pricking the several letters.

Many interesting experiments were directed towards testing traditions hitherto only combated by argument. He finds on inquiry among the best lapidaries that goat's blood is of no esteem among them for softening diamonds, as was generally supposed, and concludes, after many attempts to dissolve stone in the same medium, that "we should rather rely on a resolution of crab's eyes." That powdered glass is poison he has proved false after mixing it freely with his dogs' food for days together, and readers of Benvenuto Cellini may remember in this connection the extreme relief of that interesting scoundrel on discovering that the jailor who had been bribed to poison him with powdered diamonds, had sold the diamond supplied for the purpose, and replaced it by a sprinkling of harmless glass.

Sir Thomas Browne was no unskilled anatomist, and dissection proved to him the absurdity of many propositions then seriously upheld, as that a horse and a pigeon have no gall, and that a man hath one rib less than a woman. Common sense and observation refuted many such vulgar errors as that an elephant hath no joints, that snakes have an antipathy to ash-trees, that there is a property in basil to propagate scorpions, and that by the smell thereof they are bred in the brains of men; that bitter almonds are a cure for drunkenness, which "hath much deceived the hopes of good fellows"; that "it is good to be drunk once a month," and "that children, if taught no other language, would naturally speak Hebrew."

But he does not quite always deem it necessary to assure his readers he has proved the contrary. Sometimes the matter is left to their own judgment. "What fool almost will believe, at least, what wise man will rely on, that antidote delivered by Pierius against the sting of a scorpion—that is, to sit upon an ass with one's face towards his tail, for so the pain leaveth the man and passeth into the beast." In this category are reckoned the old prescription for a quartan ague to place the Fourth Book of Homer's "Iliad" under the head; or to administer to the patient the right eye of a hedgehog, boiled in oil and preserved in a brazen vessel. Oddly enough, the left eye of a hedgehog, fried in oil, was to procure sleep; while the right foot of a frog, wrapped in a deer's skin, was a specific for the gout. In his Commonplace book, Sir Thomas Browne has noted the following additional suggestions for treating gout: "Since you are so much unsatisfied with the many rational medicines which you say you have tried, wear shoes made of a lion's skin; try the way of transplantation; give poultices taken from the part to dogs, and let a whelp lie in the bed with you. Suffocate an eel or frog in thy wine to make thee little affected to wine. Eat partridges' eggs. Try the magnified amulet of Muffetus, of spiders' legs worn in a deer's skin; or of tortoises' legs cut off from the living tortoise and wrapped in the skin of a kid."

He despairs of refuting common superstitions by argument or proof, and merely asks, "What natural effects can reasonably be expected when for warts we rub our hands before the moon, and commit any maculated part unto the touch of the dead? When to prevent nightmare we hang up a hollow stone in our stables, and when for amulets against ague we use the chips of gallows and places of execution?"

At this time when all minds are full of the stealthy forward march of an enemy as mysterious as any of the plagues which formerly ravaged the world, a curious interest lies in Sir Thomas Browne's queries about this terrible scourge of his day—"Whether plagues be not according to the law of nature; that is, lest the world should not suffice for the number of men? Whether fire be not the greatest enemy of the plague? Whether fishes be exempt from plague? Whether the plague circulates in perpetuity, never everywhere extinct? Whether it be not equally wonderful how the plague declines, as in what manner it begins? Whether music contributes to the cure of the plague? Whether the pestiferous atoms be not animals, as Kircherus maintains!"

Strange that a physician should deem it necessary to vindicate himself with such a disclaimer as the following: "I feel not in me those sordid and unchristian desires of my profession. I do not secretly implore and wish for plagues, rejoice at famines, revolve ephemerides and almanacks in expectation of malignant aspects, fatal conjunctions, and eclipses."

But, looking beyond this disclaimer of the notorious scandals of his profession, we find in Sir Thomas Browne a truly lofty and spiritual conception of his responsibilities as a physician, and the evidence of a simple piety which might well have put to shame the enemies never lacking to any earnest seeker after truth. "Let me be sick myself, if sometimes the malady of my patient be not a disease unto me. I cannot go to cure the body of my patient but I forget my profession and call unto God for his soul."

And what shall we say to that private memorandum by a happy accident preserved among his papers? "To pray in all places where privacy inviteth; in any house, highway, or street; and to know no street or passage in this city which may not witness that I have not forgot God and my Saviour in it, and that no parish or town where I have been may not say the like."

